# Malignant pancreatic tumor other than solid pseudopapillary tumor in pediatric patients: A single-center experience

**DOI:** 10.1097/MD.0000000000027967

**Published:** 2021-12-17

**Authors:** Duon Kim, Hee-Beom Yang, Hyun-Young Kim

**Affiliations:** a Department of Premedicine, Seoul National University, College of Medicine, Seoul, Korea; b Department of Surgery, Seoul National University Bundang Hospital, Seongnam, Korea; c Department of Pediatric Surgery, Seoul National University, College of Medicine, Seoul, Korea.

**Keywords:** intraductal papillary mucinous neoplasm, mixed acinar neuroendocrine carcinoma, pancreas tumor, pancreatic neuroendocrine tumors, pediatrics

## Abstract

Pancreatic tumors, except solid pseudopapillary tumors (SPTs), are rare in pediatric patients. Herein, we report various types of pancreatic tumors in pediatric patients and review the literature regarding their treatments and prognosis.

We retrospectively reviewed the data of pediatric patients who underwent surgery for pancreatic tumors, excluding SPTs, between January 2009 and December 2019 at Seoul National University Children's Hospital. A total of 35 pediatric patients were identified as having undergone surgery for pancreatic tumors. Of these patients, 30 were excluded because the tumor was identified as an SPT.

The diagnoses of the five remaining (non-SPT) pancreatic tumors were pancreatic neuroendocrine tumor, mixed acinar neuroendocrine carcinoma, kaposiform hemangioendothelioma, and intraductal papillary mucinous neoplasm. All five patients survived; however, recurrence and liver metastasis were observed in one patient. The detailed demographics, treatments, and prognosis of each patient were reviewed.

Despite the rarity and low **i**ncidence of pancreatic tumors in pediatric patients, four types of non-SPT tumors are reported here. Hence, the possibility of these should not be overlooked, especially since the diagnosis and adjuvant treatment differ vastly between the tumor types.

## 1 Introduction

According to the National Cancer Institute Surveillance Epidemiology and End Results (SEER-17), between 1973 and 2007, the reported incidence of pediatric malignant pancreatic neoplasms was only 0.02/100,000 per year.^[1]^ Due to their rarity, the current understanding of pediatric pancreatic tumors is limited. Most pancreatic tumors in children are benign, and the prognosis is relatively good compared with that in adults; however, some types of tumors present a risk of recurrence and metastasis and should therefore not be overlooked.

There are many types of pediatric pancreatic tumors, such as pancreatoblastoma, acinar cell carcinoma, and neuroendocrine tumors. The most common is the solid pseudopapillary tumor (SPT), which is particularly common in Asians.^[2]^ Only 10% to 15% of SPT cases are malignant, and most SPTs have been reported to have a fairly good prognosis.^[3]^

Pancreatic neoplasms, SPT, and pancreatoblastoma are most commonly reported, when compared with other neoplasms. As a result, there is limited understanding of the other types of pediatric neoplasms due to a lack of information in the literature. This study reports five cases of non-SPT pancreatic tumors, including intraductal papillary mucinous neoplasm (IPMN), kaposiform hemangioendothelioma (KHE), pancreatic neuroendocrine tumor (PNET), and mixed acinar neuroendocrine carcinoma (MANEC). These types of tumors have rarely been reported in the literature, and the purpose of this retrospective study was to therefore report the rare types of pediatric pancreatic tumors identified at Seoul National University Children's Hospital between 2009 and 2019 and review their respective treatment and prognosis.

## 2 Methods

### 2.1 Patients and definitions

This retrospective, single-center study was conducted at the Seoul National University Children's Hospital. We reviewed the data of pediatric patients who underwent surgical resection for the removal of pancreatic masses between January 2009 and December 2019. The electronic schedule of pediatric surgeries during the study period was reviewed, and a total of 35 pancreatic resections were identified. Based on the pathology, 30 cases of SPT were excluded. Patient characteristics, such as initial symptoms, tumor location, diagnosis, tumor size, operation method, pathology results, complications, long-term morbidity, and adjuvant therapy, including chemotherapy and radiotherapy, as well as follow-up data were investigated. Tumor depth was assessed according to the American Cancer Society pancreatic cancer stage (T1: tumor is confined to the pancreas and is not larger than 2 cm across; T2: tumor is confined to the pancreas and is 2 to 4 cm across; T3: tumor is confined to the pancreas and is larger than 4 cm across; and T4: tumor extends beyond the pancreas into the adjacent major blood vessels).

The type of operation was selected according to the mass location, whereby pylorus-preserving pancreaticoduodenectomy (PPPD) was performed for a mass in the pancreatic head and distal pancreatectomy (DP) was performed for those in the pancreatic body. Spleen-preserving DP was performed when appropriate. The size of the mass was described in the pathology report. The surgical method was selected at the discretion of the surgeon. Early complications were defined as complications that occurred within postoperative 30 days and late complications were defined as complications that occurred after postoperative 30 days.

### 2.2 Ethics statements

This study was approved by the Institutional Review Board of Seoul National University Hospital (IRB No. 1811-053-983). The requirement for informed consent was waived due to the retrospective nature of the study. All methods used in this study were performed in accordance with the relevant guidelines and regulations.

## 3 Results

### 3.1 Patients characteristics

**Figure 1:**
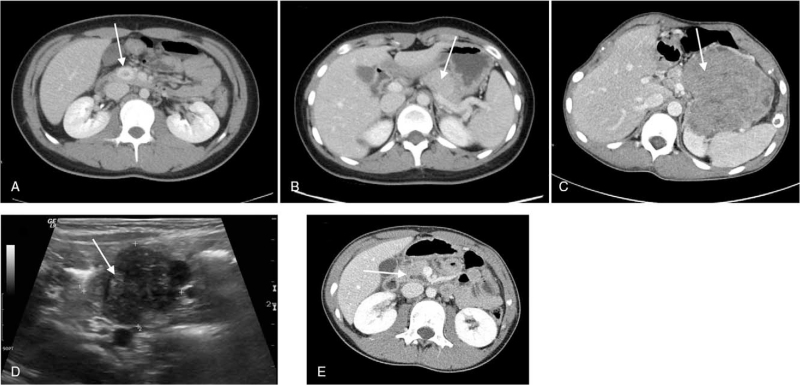
Preoperative imaging (each tumor is annotated with a white arrow). A: Patient 1, 1.9 × 1.4 cm; B: Patient 2, 3.3 × 2.9 cm; C: Patient 3, 10.0 × 9.8 cm; D: Patient 4, 2.3 × 1.9 cm; and E: Patient 5, 1.3 × 0.9 cm; Due to the small age of patient 4, computed tomography was not performed.

Five children were hospitalized with non-SPT pancreatic tumors between 2009 and 2019 (Table 1). Of these patients, four were boys and one was a girl, with a median age of 15 years (range, 1 month to 16 years). The types of tumors identified were PNET, MANEC, KHE, and IPMN. All patients, except the patient with KHE, presented with symptoms of abdominal pain. The characteristics of the five patients are presented in Table 1. Patient 1 was a 15-year-old boy who presented with symptoms of abdominal pain and loss of consciousness. His physical examination results were normal, and the laboratory findings were non-specific. Intensive diagnostic workup included abdominal computed tomography (CT) and head magnetic resonance imaging (MRI), which revealed a 2.3-cm PNET in the head of the pancreas and a 1.5-cm-sized cystic lesion, which was diagnosed as a Rathke's cleft cyst in the sella. Patient 2 was a 16-year-old girl who presented with abdominal pain and lower back pain. Abdominal tenderness and rebound tenderness were ruled out on physical examination and all laboratory findings, which included total bilirubin, AST, and serum amylase levels, were within normal ranges. Abdominal CT revealed a 3.3-cm PNET in the tail of the pancreas. Patient 3 was a 15-year-old boy, with known multiple enchondromatosis, who presented with symptoms of abdominal pain and vomiting. Physical examination revealed mild tenderness in the periumbilical area and a palpable abdominal mass. His C-reactive protein and alpha-fetoprotein levels were elevated (5.96 mg/dl and 101.9 ng/ml, respectively) on admission. Abdominal sonography and CT revealed a 15-cm pancreatic mass, and a biopsy gun revealed a malignant tumor with predominantly neuroendocrine differentiation. Patient 4 was a 1-month-old boy who was born at 38 weeks of gestation, with a birth weight of 4.1 kg, who presented with symptoms of occasional white stool. Physical examination revealed icteric sclera and whole-body jaundice. On admission, his total bilirubin/direct bilirubin level was 16.2/2.0 mg/dl and gamma-glutamyl transpeptidase level was 642 IU/L. His serology, hemolysis, and endocrine tests were non-specific. Abdominal sonography revealed a 2.1-cm pancreatic mass, whereas abdominal CT and MRI could not definitively diagnose the mass. Patient 5 was a 12-year-old boy who presented with abdominal pain and had been previously admitted to another hospital for suspected pancreatitis. Physical examination and laboratory findings were non-specific on admission. Abdominal sonography, CT, and MRI revealed a 1.3 cm IPMN in the uncinate process of the pancreas. The radiologic findings of each patient are shown in Figure 1.

**Table 1:** Patients’ characteristics.

N = 5	Case 1	Case 2	Case 3	Case 4	Case 5
Age	15 yr	16 yr	15 yr	28 d	12 yr
Sex	M	F	M	M	M
Symptoms and Signs	Abdominal Pain	Abdominal Pain	Abdominal Pain, Vomiting	White stool, Jaundice	Abdominal pain
Diagnosis	Pancreatic neuroendocrine tumor	Pancreatic neuroendocrine tumor	Mixed acinar neuroendocrine carcinoma	Kaposiform hemangioendothelioma	Intraductal papillary mucinous neoplasm
Location	Head	Tail	Tail	Uncinate process	Uncinate process
Maximal diameter (cm)	2.3	3.3	15	2.1	1.3
Distant metastasis	No	No	No	No	No
Op name	PPPD	SPDP	DP	PPPD	PPPD
Op method	Open	Robot	Open	Open	Open
Adjuvant treatment	No	No	Chemotherapy	IFN-a	No
Recurrence	No	No	Yes	No	No
Disease free survival	78 mo	4 mo	16 mo	116 mo	72 mo
Overall survival	78 mo	4 mo	33 mo	116 mo	72 mo
Survival	Yes	Yes	Yes	Yes	Yes
Tumor depth	pT2	pT2	pT3	pT3	pT1
Lymph node	No	No	No	No	No
Ki-67	Positive in <1	Focal positive	Positive in 80%	Positive in 95%	Positive in 2%
Surgical margin	Pancreatic resection margin, 3cm Pancreatic radial margin, 0.7cm Retroperitoneal margin, 0.1cm Proximal duodenal margin, 3cm Distal duodenal margin, 11cm	Pancreas parenchymal, 8cm Pancreas anterior, 0.1cm Pancreas posterior, 0.1cm	Pancreas parenchymal, 1.0cm	Pancreatic resection margin, 0cm Pancreatic radial margin, < 0.1cm Retroperitoneal margin, < 0.1cm Proximal duodenal margin, 0.6cm Distal duodenal margin, 4cm	Pancreatic resection margin, 2.0cm Pancreatic radial margin, 1.0 cm Retroperitoneal margin, 0.6cm Bile duct proximal, 0.8cm

d = days, DP = distal pancreatectomy, F = female, IFN-a = interferon alpha, M = male, mo = months, OP = operation, PPPD = pylorus-preserving pancreatoduodenectomy, SPDP = spleen-preserving distal pancreatectomy, yr = years.

### 3.2 Surgical and adjuvant treatment

All five patients underwent surgery (Table 1). Patient 1 had a tumor in the head of the pancreas and PPPD was performed. Patient 2 had a tumor in the tail of the pancreas and spleen-preserving distal pancreatectomy was performed. Patient 3 had a tumor in the tail of the pancreas and DP was performed, as well as chemotherapy as a result of spleen and transverse colon invasion. Both patients 4 and 5 had tumors in the uncinate process and PPPD was performed. Interferon alpha (IFN-α) therapy was administered to patient 4 at 1 month after the surgical resection for a duration of 4 months due to a positive resection margin.

### 3.3 Recurrence and survival

After a median period of 72 months (range, 4–116 months), all five patients survived (Table 1). However, patient 3 (who underwent DP of a MANEC) developed recurrence at postoperative 19 months. The remaining four patients survived without tumor recurrence during the follow-up period.

### 3.4 Pathology

**Figure 2:**
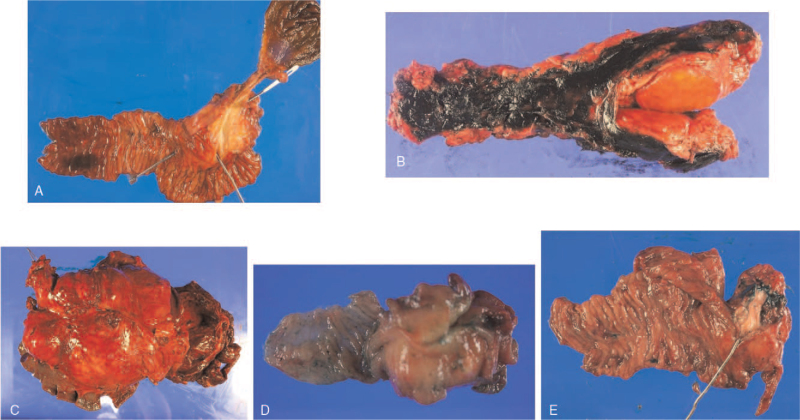
Gross picture of specimen. A: Patient 1, 2.5 × 1.8 × 1.5 cm; B: Patient 2, 2.9 × 2.6 × 2.0 cm; C: Patient 3, 15.2 × 14.9 × 8.8 cm; D: Patient 4, 2.5 × 2.0 × 1.6 cm; E: and Patient 5, 1.5 × 1.0 × 0.5 cm.

**Figure 3:**
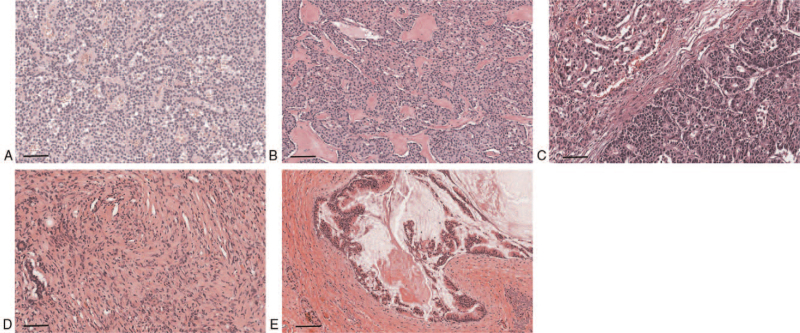
A. (Case 1) Neuroendocrine tumor: Solid nests of uniform cuboidal cells with round nuclei are observed in fibrohyalinized stroma; B. (Case 2) Same as A; C. (Case 3) Mixed acinar and endocrine carcinoma: Both neoplastic acinic and neuroendocrine cell proliferation is observed within the same tumor; D. (Case 4) Kaposiform hemangioendothelioma: In pancreas, stromal expansion with spindle cell proliferation resembling Kaposi sarcoma is observed; E. (Case 5) Intraductal papillary mucinous neoplasm with intermediate-grade dysplasia: Hyperplastic columnar cells with intermediate-grade dysplasia is observed in dilated main pancreatic duct (scale bar: 100 μm).

The tumor depth in each PNET was pT2 (Table 1). The tumor depth of MANEC and KHE was pT3, and that of IPMN was pT1. None of the tumors metastasized to the regional lymph nodes. Gross and microscopic findings of each specimen are shown in Figures 2 and 3, respectively.

### 3.5 Complications

Patient 1, who underwent PPPD, developed a late complication of acute pancreatitis (Table 2). It occurred twice, at postoperative 35 and 71 months and improved with conservative treatment. Patient 2 underwent percutaneous cavity drain placement due to fluid collection at postoperative 6 days and developed early complications of acute pancreatitis at postoperative 21 days. No complications occurred in patients 3 and 4. Patient 5, who underwent PPPD, developed early complications of duodenojejunostomy outlet obstruction at postoperative 12 days; Roux-en-Y conversion operation was performed. Patient 5 developed a late complication of acute pancreatitis 5 months after PPPD that improved with conservative treatment.

**Table 2:** Complication.

N = 5	Case 1	Case 2	Case 3	Case 4	Case 5
Diagnosis	Pancreatic neuroendocrine tumor	Pancreatic neuroendocrine tumor	Mixed acinar neuroendocrine carcinoma	Kaposiform hemangioendothelioma	Intraductal papillary mucinous neoplasm
Op name	PPPD	SPDP	DP	PPPD	PPPD
Early complication	No	Fluid collection, Acute pancreatitis	No	No	D-J stomy outlet obstruction d/t A-loop disturbance
Treatment for early complication	–	PCD insertion	–	–	Roux-en-Y conversion
Late complication	Acute pancreatitis	No	No	No	Acute pancreatitis
Treatment for late complication	Conservative care	–	–	–	Conservative care

A-loop = afferent loop, D-J stomy = duodenojejuno stomy, DP = distal pancreatectomy, PCD = percutaneous cavity drainage, PPPD = pylorus-preserving pancreatoduodenectomy, SPDP = spleen-preserving distal pancreatectomy.

## 4 Discussion

Because most pancreatic tumors in children are benign, very few cases of malignant tumors have been reported. Furthermore, while SPT and pancreatoblastoma are relatively more common in pediatric patients, MANEC, IMPN, and KHE are rare and therefore very few cases have been reported to date. Herein, we report cases of these rare tumors, analyze their respective treatment and prognosis, and review the relevant literature.

PNETs are pancreatic neuroendocrine tumor which is a subtype of gastroenteropancreatic neuroendocrine tumors, which occur in the islet cells of Langerhans.^[4]^ Neuroendocrine tumors can occur in many types of organs since neuroendocrine cells are distributed throughout various organs in the human body.^[5]^ Neuroendocrine tumors are usually more common in adults than in children. Although the incidence of neuroendocrine tumors is low in children, with approximately 2.8 cases per million, the rate is steadily increasing.^[6]^ Pancreatic origin is considered a factor in poor prognosis.^[7]^ PNETs are classified as either functional or nonfunctional and are mostly malignant depending on the production and secretion of pancreatic endocrine hormones. Approximately 10% of PNETs are functional, with insulinomas being the most common.^[8]^ PNETs can also be classified as either well differentiated or poorly differentiated, with most being well differentiated and nonfunctional. The Ki-67 index of neuroendocrine patients reported in this study was low, indicating that the tumors were well differentiated. The majority of PNETs are sporadic (90%) but can also be associated with genetic syndromes, such as multiple endocrine neoplasia type 1.^[8]^

MANEC is mixed acinar neuroendocrine carcinoma, which is composed of both acinar cells and neuroendocrine cells. The pancreas is composed of both exocrine and endocrine components: the exocrine component is composed of ductal and acinar cells and the endocrine component is composed of endocrine cells. Acinar cell carcinoma and neuroendocrine carcinoma are rare types among pancreatic neoplasms.^[9]^ To differentiate between MANEC and acinar cell carcinoma, more than 30% of the tumor must comprise neuroendocrine cells.^[10]^ However, very few cases of MANECs have histologically distinct acinar and neuroendocrine areas, with most cases representing a uniform cell population. MANECs are mostly located in the head of the pancreas. The prognosis of MANEC has been reported to be poor with a median survival of 12 months, which is similar to that of acinar cell carcinoma. Furthermore, chemotherapy and radiotherapy are considered ineffective.^[11]^ The patient with MANEC (patient 3) survived for 2 years during the follow-up period. However, a liver metastasis and tumor recurrence were found 19 months postoperatively. DP was performed for the tumor in the pancreatic tail and spleen of this patient, with the final pathological diagnosis of the tumor being MANEC in the pT3 stage. A previous case report of this patient has been published^[12]^; however, during the additional follow-up period after the case report was published, we found liver metastasis and tumor recurrence. Sixteen months after DP, a metastasis was found in segment 7 of the liver and tumorectomy was subsequently performed. Three months later, the tumor recurred in the pancreatic tail and another DP was performed. In a review of 21 MANEC cases published in English, 10 of 21 cases had distant metastases, with 9 of those 10 being liver metastases.^[13]^ MANEC is a rare but aggressive tumor with a risk of metastasis and tumor recurrence. Therefore, accurate diagnosis and complete resection are important.

KHE is kaposiform hemangioendothelioma. Usually KHE is a locally aggressive vascular tumor that can infiltrate other tissues.^[14]^ KHE is a rare tumor known to occur in infants or young children, with an incidence of approximately 0.07/100,000 children per year.^[15]^ The pancreas is an extremely rare location for a KHE in a child, with only nine reported cases between 1973 and 2015.^[14,16]^ KHE is commonly associated with the Kasabach–Merritt phenomenon,^[17]^ whereby a retrospective study of 107 patients with KHE between 1991 and 2009 reported that the phenomenon developed in 71% of the patients.^[18]^ Thrombocytopenia caused by platelet trapping and consumptive coagulopathy can be observed in the Kasabach–Merritt phenomenon.^[19,20]^ However, patient 4 in this study, who was a 1-month-old infant with KHE, did not develop the Kasabach–Merritt phenomenon. Patients with hemangioendothelioma (HE) generally show signs and symptoms of direct bilirubin increase, obstructive jaundice, yellow urine, and white stool. As previously described, patient 4 presented with jaundice and white stool symptoms. Biliary obstruction in patients with HE can be resolved by palliative surgery (biliary drainage) or the Whipple procedure and postoperative IFN administration if the tumor size is particularly large.^[16]^ Patient 4 showed improvement received IFN-α as adjuvant treatment after PPPD. KHE is benign and has a good prognosis. Patient 4 survived without recurrence during the 10-year follow-up period.

IPMN is intraductal papillary mucinous neoplasm, which is a tumor that occurs mainly in older adults aged 70–80 years that shows proliferation of mucin-producing cells in a papillary pattern. Most IPMNs occur in the pancreatic head and can be treated with complete surgical resection.^[1]^ Most patients with IPMNs present with recurrent pancreatitis. To date, only two cases of IPMN have been reported in children.^[21]^ The first reported pediatric IPMN case was of a 14-year-old patient with epigastric pain and increased pancreatic enzyme secretion.^[22]^ CT and ultrasound examinations revealed pancreatic duct dilation without a definitive lesion. One year later, a fisheye appeared on the major and minor papilla and a lesion was found on the pancreatic head; PPPD was subsequently performed. The patient with IPMN reported in this paper, similar to most patients with IPMN, had symptoms of recurrent pancreatitis and abdominal pain. Cystic lesions and main pancreatic duct dilation were observed. IPMNs are premalignant lesions, and the 3-year survival rate after surgical resection is 60% to 80%. However, in the case of invasive cancer, the survival rate decreases to 21%.^[21]^ To the best of our knowledge, only three pediatric IPMN cases have been reported to date; however, the fact that IPMN can occur in children and may be a cause of pancreatitis should be considered during the diagnostic process.

This study is a retrospective case series with a small number of patients and there are therefore several limitations. Due to the small number of patients and lack of a control arm, statistical analysis and meaningful results could be lacking. Further, we thought that all pancreatic tumors except SPT in the pediatric population were included during the study period, but there could be missed cases due to the retrospective nature of the study.

In this study, we report five pediatric cases of PNET, MANEC, KHE, and IPMN who all underwent surgical resection. Four patients had complications of acute pancreatitis, with two early complications and two late complications. All five patients survived throughout the follow-up period, although one had tumor recurrence and liver metastasis. Pancreatic tumors are rare in pediatric patients generally, and while these four specific types of pediatric pancreatic tumors are also rare and have a low incidence, they should not be overlooked in the diagnostic process because they each have different prognoses and treatments.

## Acknowledgment

The authors thank Clinical Professor Jaemoon Koh, Department of Pathology, Seoul National University, for the kind description of tumor histology.

## Author contributions

**Conceptualization:** Duon Kim, Hee-Beom Yang, Hyun-Young Kim.

**Data curation:** Duon Kim, Hee-Beom Yang.

**Formal analysis:** Duon Kim, Hee-Beom Yang.

**Investigation:** Duon Kim, Hee-Beom Yang.

**Methodology:** Duon Kim, Hee-Beom Yang, Hyun-Young Kim.

**Resources:** Duon Kim.

**Supervision:** Hyun-Young Kim.

**Validation:** Duon Kim, Hee-Beom Yang, Hyun-Young Kim.

**Visualization:** Duon Kim, Hee-Beom Yang.

**Writing – original draft:** Duon Kim, Hee-Beom Yang.

**Writing – review & editing:** Duon Kim, Hee-Beom Yang, Hyun-Young Kim.
